# Integrated data on the taxon and morpho-specific year-round diet and endozoochorous seed dispersal by the world’s largest grouse, the Capercaillie *Tetrao urogallus*

**DOI:** 10.1038/s41597-025-05811-1

**Published:** 2025-08-27

**Authors:** Joanna Czarnecka, Dorota Merta, Janusz Kobielski, Jerzy Karg, Grzegorz Orłowski

**Affiliations:** 1 Department of Botany, Mycology and Ecology, Institute of Biological Sciences, Maria Curie-Skłodowska University, 19 Akademicka Str., 20-033 Lublin, Poland; 2 Institute of Biology and Earth Sciences, University of the National Education Commission, 2 Podchorążych Str., 30-084 Kraków, Poland; 3 Ruszów Forest District, 2 Leśna Str., 59-950 Ruszów, Poland; 4 Institute of Technology and Life Sciences – National Research Institute, Falenty, Al. Hrabska 3, 05-090 Raszyn, Poland

**Keywords:** Forest ecology, Ecosystem ecology

## Abstract

Recent developments in molecular methods for fecal analysis to assess the food habits (scatology) of herbivores have been controversial in terms of accurately identifying which specific plant parts were consumed and quantifying the amount of food ingested. To address this critical issue, traditional methods should be used to describe a multi-component plant diet that expresses the contribution of taxon-specific morphologically differentiated plant parts. Here we present quantitative data from our original high-resolution taxon- and morpho-specific dietary study based on cuticle microhistological analyses of food remains from the feces of the Capercaillie *Tetrao urogallus*. By providing integrative quantitative dietary data based on the functional classification of different plant parts representing 49 types of food items belonging to four major food categories (seed and fruit remains, leaves, moss fragments, and other plant material like stem or inflorescence fragments), intact seeds, arthropods, and mineral particles (grit/gastroliths), our dataset has potential applications in studies of the diet, endozoochorous seed dispersal capabilities, and the reintroduction biology of gallinaceous birds. We highlight that Galliformes consume different fragments of the same plant taxa with varying intensity throughout the year. Therefore, qualitative methods based on the frequency of occurrence of individual plant taxa using fecal DNA do not provide actual quantitative data on the diet composition in terms of ingested specific plant parts. Hence, a parallel microhistological analysis is essential to describe the full spectrum of the diet of herbivores consuming various plant fragments.

## Background & Summary

A thorough analysis of an animal’s diet, based on the breakdown of structurally different parts of the same prey type, is a critical starting point for accurately describing plant-animal interactions in terms of the selection of specific food resources. Such a detailed assessment of the types of food consumed is essential for unambiguously linking the requirements of a given species with its living environment and the intrinsic mutual plant-animal interactions. Such analysis is also vital for formulating evidence-based planning and adaptive management strategies for populations of animals that are of conservation concern. A comprehensive description of diet is particularly important for herbivores that consume varying amounts of distinct parts of the same plant taxa, including seeds, shoots, stems, leaves, buds, flowers, fruits, or roots. Importantly, all these plant parts differ significantly in nutritional traits, energy content, and digestibility^1–4^.

Recent developments in molecular scatology are considered low-cost, time-efficient, and capable of providing high taxonomic resolution dietary data^5–7^. However, this method cannot determine which specific plant parts were consumed or quantify the amount of food ingested. Most importantly, while the findings of dietary studies derived from molecular analysis are taxonomically informative, the lack of functional classification of the plant food items renders them controversial and prevents a comprehensive description of the diet. Therefore, these findings should be interpreted with caution. Consequently, there is a need to employ methods that enable the distinction of taxon-specific morphologically differentiated plant parts that were consumed.

Gallinaceous birds are examples of herbivorous animals that consume various plant parts throughout the year^8–12^. Due to their predominantly sedentary lifestyle and habitat in seasonal environments – particularly in the Northern Hemisphere, where food resources fluctuate significantly in a phenological pattern – the dietary description of Galliformes necessitates consideration of not only the species composition (the frequency of occurrence of specific taxa revealed by dietary studies using a fecal metabarcoding approach^5–7^; of the ingested prey or plant taxa but also a numerical representation of the entire spectrum of morphologically distinct functional food components of individual plant taxa^8–12^. Notably, both forest and farmland Galliformes can ingest leaves, seeds, and other plant parts from various species of trees, including both monocots and dicots, depending on the availability of these types of plant food items in their habitat^8–13^.

Regarding plant–herbivore interactions, an equally important issue is that Galliformes can serve as significant agents of long-distance seed dispersal (LDD) operating at a relatively small – landscape – scale^14^. This occurs through endozoochory, internal dispersal in the animal’s gut^15^, in both natural (forest) and human-modified agricultural settings^16–19^. However, detailed data linking the numerical dietary specificity/correlates (i.e., the quantified variety of various plant food items) to the presence of intact seeds after passage through the gastrointestinal tract (i.e., recovered from feces) are scarce for Galliformes^16,18^ and warrant in-depth studies, particularly in the context of plant invasiveness and the functioning and recovery of plant communities. Notably, prior studies indicate that Galliformes can act as both seed predators and seed dispersers, with this distinction relating to the traits of the consumed food or plant, suggesting a continuum between these two services^17,19^. Specifically, only a negligible number of dry-fruited plant seeds (e.g., agricultural weeds) pass through the digestive system intact. For instance, merely 0.3% of ingested *Amaranthus retroflexus* seeds resist digestion in the Grey Partridge *Perdix perdix*^20^, while the majority of seeds from fleshy-fruited plants (such as *Vaccinium myrtillus*) can remain intact and viable after passage through the digestive tract^18,19^.

Importantly, over the past few decades, native gallinaceous birds have experienced a significant decline in population across both natural (forest) and semi-natural (agricultural) habitats in the Northern Hemisphere^21–26^. An example of such a process is the world’s largest grouse, the Western Capercaillie *Tetrao urogallus*, with adult females weighing between 1.4 and 2.5 kilograms and adult males weighing between 3.0 and 6.5 kilograms^27^. The Capercaillie is a rare sedentary forest species that prefers large old-growth natural forests occurring in certain boreal and mountainous regions of Europe^21,22,28^. In conservation, the Capercaillie is considered an iconic and umbrella species symbolizing wild forests^29–31^.

In most of its range, the Capercaillie has been declining dramatically during the last few decades^21,25,32–36^. Although the species is not considered to be globally threatened^37^, many local populations in Central and Western Europe have been extinct, and the remaining small and isolated populations are threatened^21,38^. Only in the boreal forests of Fennoscandia and western Russia, Capercaillies still occur in considerable numbers. However, also in these regions, a population decline has been observed^32,39,40^.

The most important threats to Capercaillies are unfavorable changes in the forest structure (habitat loss, habitat degradation, forest fragmentation) due to human land use^28,32,41–43^, the effects of predators^22,23,44–48^, weather conditions^24,49^, hunting and human disturbance^50–53^, and a small population size and genetic isolation^33,34,36,54,55^. To restock or re-establish local populations of Capercaillies, the release of birds reared in captivity or captured in the wild has become a common conservation strategy in many European countries, including Poland^35,56–58^.

Although food availability is not considered a major driver of the population decline in the Capercaillie and other species of forest grouse – primarily due to their folivorous diet and reliance on abundant plant resources – and despite the well-documented diet of these birds, the recent developments in molecular methods of fecal analysis (scatology) for assessing the composition of the diet of grouses^5–7^ do not facilitate the distinction of the different plant fragments consumed. Therefore, there is a need to employ methods that enable taxon- and morpho-specific identification of plant food.

**Fig. 1:**
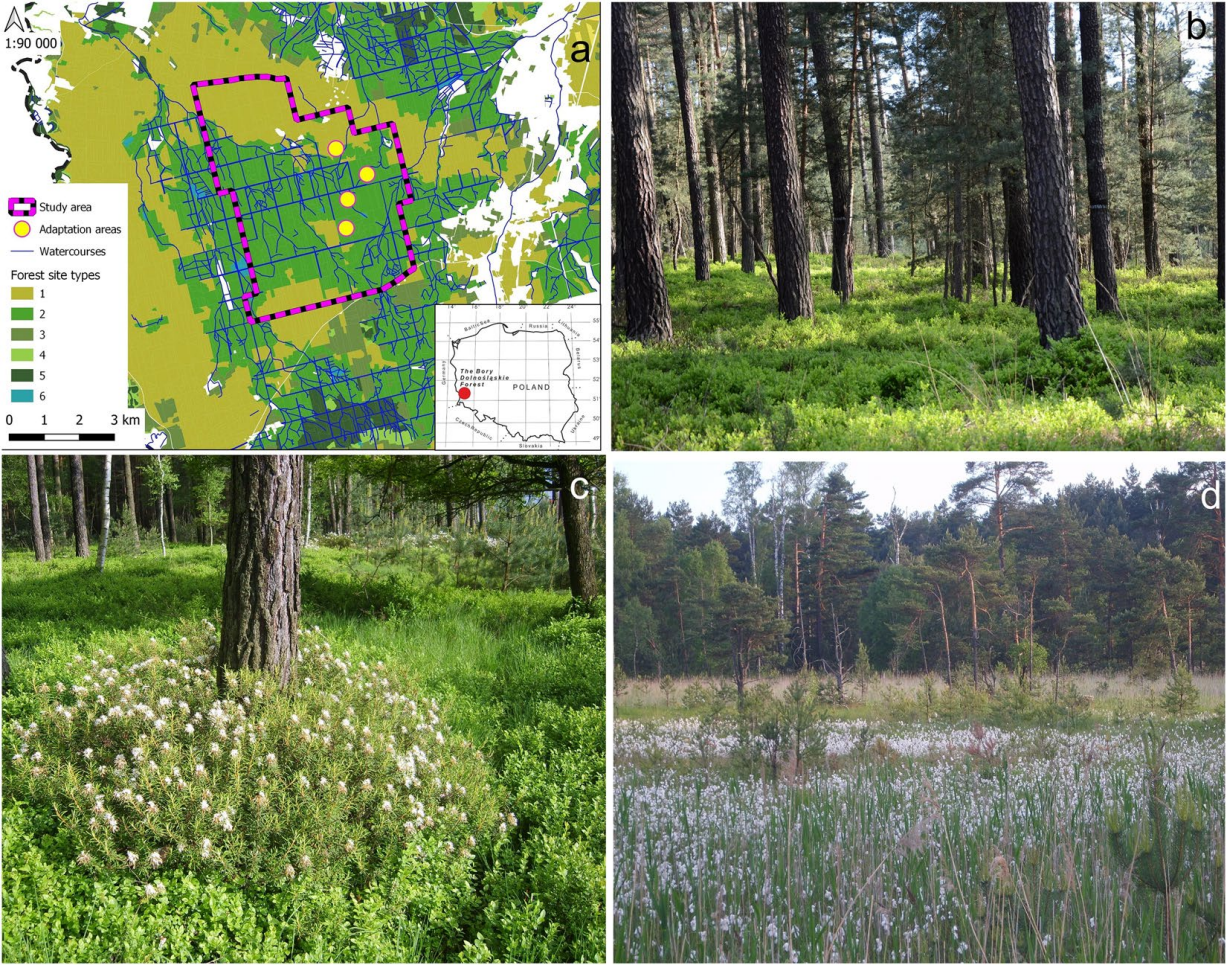
The area of Capercaillie reintroduction in the Ruszów Forest District, Bory Dolnośląskie (Lower Silesian Forest). Forest habitat types (abbreviations on the map – a): 1 – mesic coniferous; 2 – moist coniferous and moist mixed coniferous; 3 – mesic mixed coniferous; 4 – mesic broadleaved and mesic mixed broadleaved; 5 – moist broadleaved and moist mixed broadleaved; 6 – swamp coniferous and swamp mixed coniferous. The photos refer to following forest habitat types: (b) mesic coniferous (© Dorota Merta), (c) moist coniferous (© Janusz Kobielski) and (d) swamp coniferous (© Janusz Kobielski).

**Fig. 2:**
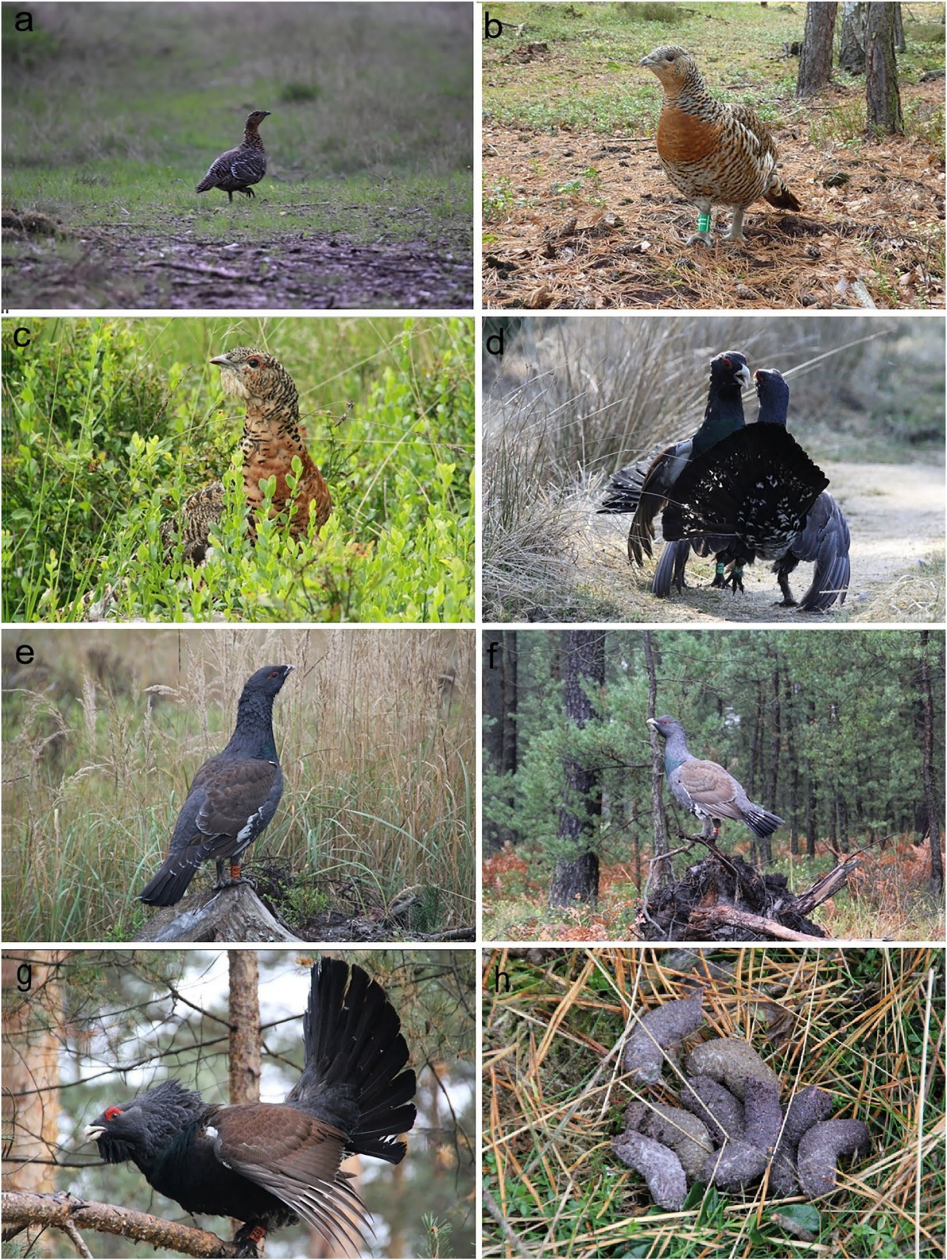
Major habitat types used by Capercaillies in the Bory Dolnośląskie forest. (**a**) The unringed hen on the forest floor showing the distraction behavior may suggest the presence of chicks. May 2020 (© Ziemowit Rejniak). (**b**) Ringed hen on a Scots pine *Pinus sylvestris* forest floor, Nov. 2010 (© Jakub Furtek). (**c**) Hen on a blueberry *Vaccinium myrtillus-*dominated forest floor, Aug. 2009 (©Janusz Droś). (**d**) Cocks during an aggression event on a forest dirt road, Mar. 2017 (© Janusz Kobielski). (**e**) Cock in a clear-cut plant community (Pine forest) with bush grass *Calamagrostis epigejos*, a plant species with clonal growth, and blueberry *Vaccinium myrtillus*. Oct. 2010 (© Janusz Kobielski). (**f**) Cock in the habitat of a naturally regenerating plant community with Scots pine *Pinus sylvestris*. Forest floor dominated by bracken fern *Pteridium aquilinum*. Nov. 2010 (© Dorota Merta). (**g**) Cock on Scots pine *Pinus sylvestris*; needles of this plant are a staple diet item in the autumn-winter period. Oct. 2011 (© Janusz Kobielski). (**h**) An aggregation of droppings, Jul 2009 (© Dorota Merta).

**Fig. 3:**
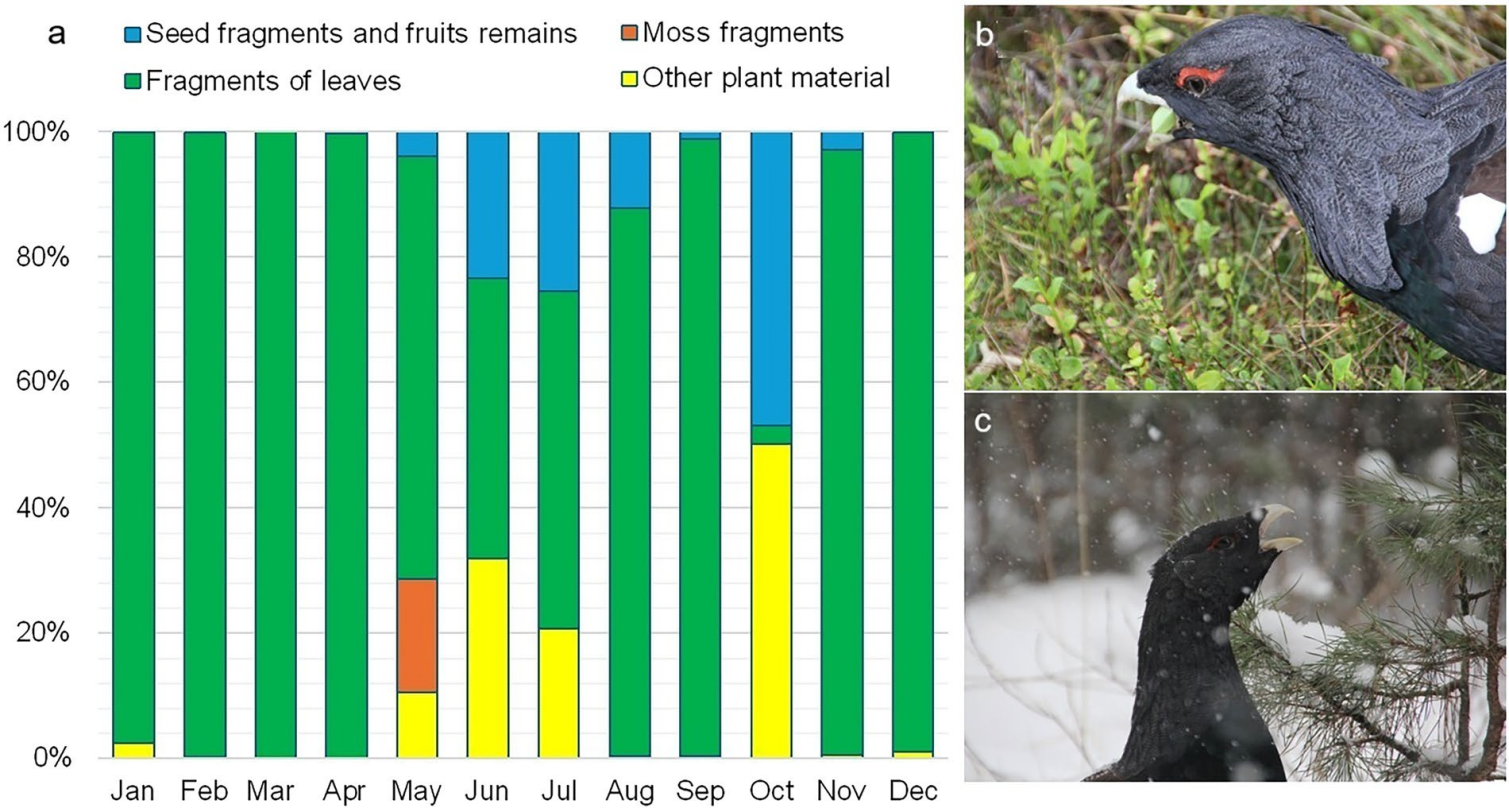
Diet of the Capercaillie. (**a**) The structure of diet showing the proportion of the four main plant components in successive months. (**b**) The individual feeding on *Vaccinium myrtillus* leaves (© Dorota Merta). (**c**) The individual feeding on Scots pine needles (© Dorota Merta).

In this paper, we present raw quantitative data from our original dietary study of a reintroduced population of Capercaillies in the extensive Scots pine *Pinus sylvestris* forest region of Bory Dolnośląskie in southwestern Poland (Fig. 1). Our dataset includes a functional division of plant food categorized by taxon- and morpho-specific characteristics, determined through cuticle microhistological analyses of plant remains found in feces. Additionally, our dataset integrates high-resolution dietary data with the quantity of intact seeds recovered from droppings sampled throughout the year (Table 1). The dietary data presented in Table 1 were derived from the examination of 0.1-g samples derived from 80 droppings, with a total mass of 99 grams, sampled from various habitat types that differ in the floristic composition and represent the primary foraging grounds used by the focal species (Fig. 2). We identified 158,812 distinct food items from 49 different types, categorized into four major food categories or plant parts: leaves, buds, inflorescences, and fruits. These items primarily consisted of base plant taxa (Table 2), including *Pinus sylvestris*, *Vaccinium myrtillus*, *Vaccinium vitis-idaea*, and several monocotyledon taxa, predominantly *Poaceae* (grasses). The monthly dietary variations per individual 0.1 g sample from individual dropping are presented in Table 1. The data show the key seasonal shifts in the plant diet related to the phenology and availability of specific plant taxa, e.g., *Vaccinium* ssp. dominance in summer and a monodiet based on *Pinus sylvestris* needles in autumn and winter (Table 1, Fig. 3).

**Table 1:** Results of the microhistological dietary analysis of individual Capercaillie droppings sampled in subsequent months.

(a) January–March																				
Food item/diet characteristic	Jan										Feb					Mar				
	1	2	3	4	5	6	7	8	9	10	1	2	3	4	5	1	2	3	4	5
**Total weight of droppings (g)**	1.504	1.063	1.309	0.870	1.280	0.945	1.126	2.093	1.627	1.274	1.450	1.427	2.600	2.760	1.091	1.280	1.885	1.165	1.614	0.833
**Seeds (number per one dropping)**																				
*Vaccinium myrtillus*					1								1							
*Vaccinium vitis-idaea*					1															
Subsample 0.1 g																				
**Seed fragments and fruits remains**																				
Cereal fragments	23	3									5	2		14	1					
*Polygonum* seed shells						2														
*Vaccinium vitis-idaea* seed shells															1					
**Fragments of leaves**																				
*Pinus sylvestris* needles	1896	1567	2006	2296	1528	1679	2056	2269	2467	2086	2427	2483	2084	2623	2333	2443	2378	2500	2337	2268
*Pinus sylvestris* scale leaves	172	89	252	312	107	76	259	187	320	201	398	175	213	264	273	211	200	202	185	188
*Vaccinium myrtillus* leaves																				8
Unidentified Dicotyledonous leaves	6													5	2		2			
**Moss fragments**																				
Moss leaves								7												
**Other plant material**																				
*Pinus sylvestris* cone fragments		14			68	20		3		40										
Cereal husks		130			28	80				76				8	24					
Other Poaceae husks					3															
Unidentified plant material		30	40	5																
**Chitin fragments**													1							

(b) April–June																									
Food item/diet characteristic	Apr					May										Jun									
	1	2	3	4	5	1	2	3	4	5	6	7	8	9	10	1	2	3	4	5	6	7	8	9	10
**Total weight (g)**	1.217	1.050	0.744	1.084	1.225	1.162	1.379	1.428	2.801	2.571	2.032	2.096	2.999	2.284	3.848	0.618	0.563	0.582	0.886	0.614	0.266	0.650	1.406	0.715	0.552
**Seeds (number in the whole dropping)**																									
*Vaccinium myrtillus*																1									
*Vaccinium vitis-idaea*	10	7			1					2															
*Vaccinium myrtillus* unripe																16						1			
**Subsample 0.1 g**																									
**Seeds (number per subsample)**																									
*Vaccinium vitis-idaea*	4	1																							
**Seed fragments and fruits remains**																									
*Aronia* sp. pulp						1554							498												
*Aronia* sp. seed shells						78			3				30												
*Carex pilulufera* seed shells																510	949		182	745	688	747	283		619
Cereal fragments						25							1												
*Vaccinium myrtillus* seed shells																								2	
*Vaccinium myrtillus* fruit fragments																									
*Vaccinium vitis-idaea* fruit fragments	11	25																							
**Fragments of leaves**																									
*Pinus sylvestris* needles	2469	1692	3135	2578	2914	354				322	2579			1163	119			2109	1232				1452		
Pinus sylvestris scale leaves	197	36	71	214	135	15				21	31			225	54			215	22				125	1703	
*Picea* sp. needles												32													
*Larix* sp. needles													152												
*Vaccinium myrtillus* leaves							282	343				1503	19	45	30	627			11		10	1	49	34	1
*Vaccinium vitis-idaea* leaves	57	297			15										57	17		8	60	23	7	24			
*Vaccinium* sp. leaves																38									
*Fagus sylvatica* leaves										1000															
*Carex pilulifera* leaves and stems																571	190		2	65	53	44	14		105
Poaceae leaves							76	23		27					19									73	
Unidentified Dicotyledonous leaves						5			776			8			487			13		18	4	8		109	
**Moss fragments**																									
Moss leaves							875	717		58		9	1		7										
Moss sporangia fragments							19																		
Stem/rhizoids fragments																									
of *Polytrichum* sp. and *Marchantia* sp.							473	247				23		5	71										
**Other plant material**																									
*Vaccinium* sp. stamen fragments												166		3	38										
*Vaccinium* sp. stem fragments												2													
Cereal husks						50		17	515	34		4	129	60	26										
*Carex pilulifera* inflorescence fragments																264	991			1065	914	744	185		922
*Carex pilulifera* perigynium fragments																44	368		4	98	233	253	35		353
Unidentified plant material							167	167	7	85		44	4												
**Chitin fragments**										29															

(c) July–September																				
Food item/diet characteristic	Jul										Aug									
	1	2	3	4	5	6	7	8	9	10	1	2	3	4	5	1	2	3	4	5
**Total weight (g)**	1.547	0.675	1.410	1.401	0.883	0.497	1.508	0.749	1.179	1.108	0.366	0.257	0.289	0.909	0.491	0.582	0.419	0.332	0.417	1.089
**Seeds (number in the whole dropping)**																				
*Vaccinium myrtillus*	215	144	868	242	267	188	163	432	519	148	83	66	34		11			5		
*Vaccinium myrtillus* unripe	149	121																		
*Juncus* sp.			1			10					3	3	5		3					
*Betula* sp.						1														
Subsample 0.1 g																				
**Seeds (number per subsample)**																				
*Vaccinium myrtillus*	23	52	151	69	58	78	43	89	130	48	68	54	29		6			4		
**Seed fragments and fruits remains**																				
*Carex* sp. seed shells													4							
Cereal fragments																			4	
*Juncus* sp. seed shells											1	5	4		1					
*Juncus* sp. capsula fragments					17	28														
*Vaccinium myrtillus* seed shells	691	672	214	338	241	397	419	604	684	270	335	525	213	2	36	2	3	106	2	2
*Vaccinium myrtillus* fruit fragments	15	21																		
**Fragments of leaves**																				
*Pinus sylvestris* needles	1177	825	109	1473	377	146	929	941	221	253	788	1840	786	2023	1942	2202	2030	980	1919	2187
*Pinus sylvestris* scale leaves	5										55	26	17	32	38	167	164	32	180	199
*Vaccinium myrtillus* leaves	13	60	7	47	6	134	7	13	34	169	142	109	48	40	16			1		
*Vaccinium vitis-idaea* leaves									3											
Poaceae leaves						11		2										252	3	
*Juncus* sp. leaves						31														
Other leaves	844	540	438	467	390	48	2	7			155	12	6		12		31		10	2
**Moss fragments**																				
Moss leaves				7									15							
**Other plant material**																				
Cereal husks	19																			
*Calamagrostis* sp. husks										6										
*Poa compressa* husks	3		2	23	357	638	331	538	913	233										
Other Poaceae husks														16				37		
*Poa compressa* inforescence fragments					34	307	19	32	259	46										
**Chitin fragments**									5											
**Stones**		158				74														

(d) October–December															
Food item/diet characteristic	Oct					Nov					Dec				
	1	2	3	4	5	1	2	3	4	5	1	2	3	4	5
**Total weight (g)**	0.731	0.857	1.041	1.650	1.157	1.430	0.624	1.796	1.295	1.062	2.235	2.092	1.191	1.259	1.161
**Seeds (number in the whole dropping)**															
*Vaccinium myrtillus*	2	6	3		3						6	38	3	1	1
*Vaccinium vitis-idaea*						6		55	94	150					
*Setaria pumila*		1													
**Subsample 0.1 g**															
**Seeds (number per subsample)**															
*Vaccinium myrtillus*	2	3	1									8		1	
*Vaccinium vitis-idaea*						3		7	17	21					
**Seed fragments and fruits remains**															
Cereal fragments	732	755	540	603	626										
*Vaccinium myrtillus* seed shells	2		3		4							3	2	12	
*Vaccinium vitis-idaea* seed shells						13	2	55	32	151					
**Fragments of leaves**															
*Pinus sylvestris* needles	54	25	22	32	78	2119	1752	1618	1147	666	1464	821	1624	913	1930
*Pinus sylvestris* scale leaves						162	194	59	13		290	64	155	106	406
Other unidentified needles												20			
*Vaccinium myrtillus* leaves						2									
*Vaccinium vitis-idaea* leaves								6		79					
*Vaccinium* sp. leaves									30	315					
*Fagus sylvatica* buds												790		217	
Poaceae leaves								6	74	18					
Other leaves	1					17	4	37	26		3				
**Other plant material**															
*Pinus sylvestris* cone fragments													13		
Cereal husks	606	712	773	672	744			34	4	16			12		
Other Poaceae husks									2						
Twig fragments											8	43	21		
**Chitin fragments**									4						
**Stones**											72	57	6	212	

The table includes the number of identified food items categorized by major dietary components (in bold) and the number of intact seeds found in a subsample (0.1 g) and in the whole individual droppings. The total number of seeds in the dropping is the sum of the number of seeds in the subsample and in the remainder of the dropping. Only food items recorded in a given subsample are included in the table. A complete list of food items is available in the Zenodo data repository^76^. Fruits of *Vaccinium myrtillus* do not leave much residue, but they stain droppings purple; additionally, the red coloration of some droppings suggests the consumption of *Vaccinium vitis-idaea* fruits.

**Table 2:** Basic information on the ecology of plant species whose fragments or seeds and other plant material were identified in the Capercaillie droppings.

Species/Taxa	Flowering time^78^	Fruit availability^79^	Fruit type/Seeds^80^	Diaspore (dispersal unit) type^80^	Dispersal mode^15^	Life form^15^
*Betula* sp.	April-May	April-October	Non-fleshy fruit	Fruit	Anemochory	Tree
*Calamagrostis* sp.	June-August	June-October	Non-fleshy fruit	Fruit	Anemochory/ no dispersal adaptations	Herbaceous plant
*Carex pilulifera*	April-June	April-August	Non-fleshy fruit	Fruit	Myrmecochory	Herbaceous plant
*Fagus sylvatica*	April-May	June-October	Non-fleshy fruit	Fruit	Endozoochory/ Vertebrate hoarding	Tree
*Juncus* sp.	May-September	June-September	Fruit with upright aperture	Seed	No dispersal adaptation	Herbaceous plant
*Larix decidua*	April-May	April-November	Seed (gymnosperme)	Seed	Anemochory	Tree
*Picea abies*	May	April-December	Seed (gymnosperme)	Seed	Anemochory	Tree
*Pinus sylvestris*	May	March-December	Seed (gymnosperme)	Seed	Anemochory	Tree
*Poa compressa*	June-August	May-June	Non-fleshy fruit	Fruit	No dispersal adaptation	Herbaceous plant
*Polygonum* sp.	July-October	July-October	Non-fleshy fruit	Fruit	No dispersal adaptation/ Freshwater hydrochory	Herbaceous plant
*Setaria pumila*	July-September	July-October	Non-fleshy fruit	Fruit	Epizoochory	Herbaceous plant
*Vaccinium myrtillus*	May-June	June-September	Fleshy fruit	Fruit	Endozoochory	Shrub
*Vaccinium vitis-idaea*	May-August	July-November	Fleshy fruit	Fruit	Endozoochory	Shrub

For taxa classified to the genus level, summary data are provided for the most abundant species in the Polish flora.

Source of information is given in the first raw of the table.

**Fig. 4:**
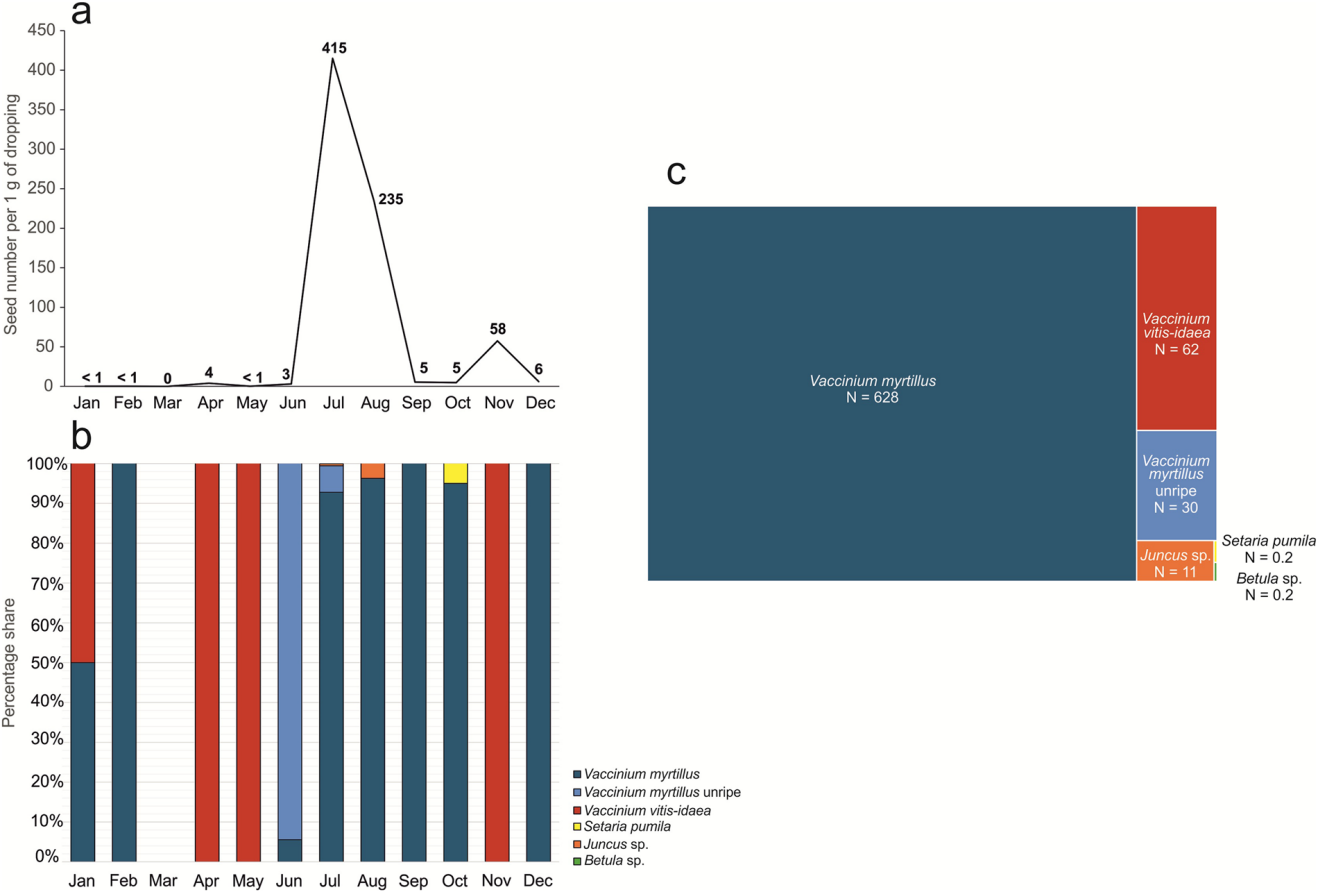
Seeds found in Capercaillie droppings in subsequent months. (**a**) Total number of seeds was calculated per 1 g of droppings. (**b**) Proportion of seeds in subsequent months. Note the significant differences in the total number of seeds recorded in individual months, which was used as the basis for evaluating the species composition of the seed pool. (**c**) Proportion of seeds representing different taxa in the total seed pool. N – total amount of seeds in feces after converting the amount in the samples from subsequent months to 1 g.

We recorded 4 091 intact seeds, of which 287 were unripe (Fig. 4). The majority of intact *Vaccinium myrtillus* seeds (92% of the total) were collected in summer (July), while intact *Vaccinium vitis-idaea* seeds (94%) were predominantly found in autumn (November) (Fig. 4). Notably, seeds from dry-fruited plants were also discovered, including 25 seeds of *Juncus* sp. as well as single seeds of *Betula* sp. and *Setaria pumila* (Table 1, Fig. 4).

Our dataset also includes information on the number of stones, which are presumably used as grit to grind hard food in the gizzard (Table 1), as well as minor animal food sources (Table 3), which are likely ingested to a greater extent by chicks and adolescent Capercaillies^59–61^.

**Table 3:** Arthropod components (identified to taxon, family, or order) in the diet of Capercaillies determined based on the analysis of 80 droppings sampled in May 2014 (*n* = 38) and June 2014 (*n* = 42).

#sample (code)	May		#sample (code)	June	Number of individuals
	Food item/component	Number of individuals		Food item/component	
0–5	Araneae	3	0–1	Coleoptera (uniden.)	1
	Curculionidae	1	0–7♀	Araneae	1
	Coleoptera (uniden.)	1	0–13♂	Pentatomidae	1
0–8	subsample 1		0–20♂	Araneae (one subsample)	1
	*Agriotes* sp. (Elateridae)	1	0–22	*Aleochara* sp.	1
	Curculionidae	1		Ichneumonidae	1
	Coleoptera (uniden.)	1		Araneae	1
	*Necrophorusb* sp.	1	0–25	Araneae	>3
	subsample 2			Coleoptera (uniden.)	1
	Araneae	1	0–31	Araneae	2
	Curculionidae	1		*Formica* sp.	1
	Coleoptera (uniden.)	1		Curculionidae	1
0–10	Araneae	1		Coleoptera (uniden.)	1
0–14	Araneae	1		Small stone (grit)	1
0–17	Curculionidae	1		Coleoptera (uniden.)	1
0–25	Coleoptera (uniden.)	1	0–34	Coleoptera (uniden.)	1
0–26	Coleoptera (uniden.)	1	0–36	subsample 1	
	Ceutorrhynchus	1	0–40♀	*Myrmica* sp.	>40
0–27	Araneae	2		Araneae	2
0–29	*Myrmica* sp.	1		Carabidae	1
				Coleoptera (uniden.)	2
				subsample 2	
				*Myrmica* sp.	10
				Coleoptera (uniden.)	1
				subsample 3	
				*Myrmica* sp.	23
				Coleoptera (uniden.)	1
				Araneae	1

The table summarizes only the data for droppings containing arthropod prey items; droppings with plant remains were disregarded. A subsample refers to one dropping selected from a larger group of droppings.

Our study show that, despite the supplementary feeding (a small amount of cereal mixture, mainly oats, and fruits, mainly chokeberries), the Capercaillies consumed primarily natural plant food throughout the year (Table 1). This indicates the low importance of supplementary food and the very good preparation of birds from Capercaillie breeding centers (Kadzidłowo, Wisła, Leżajsk) for life in the natural environment. On the other hand, maintaining permanent feeding sites increases the chance that the birds will remain in adaptive areas protected from predator pressure until the beginning of natural dispersion, which significantly reduces their mortality in the first critical weeks after release^47,48^. Moreover, observations indicate that Capercaillies are eager to use supplementary feeding in winter when there is a thick snow cover.

## Methods

### Study area

The material used in this study was collected as part of the Capercaillie reintroduction program in the Bory Dolnośląskie Forests, which has been implemented by the Ruszów Forest District since 2009 and is currently being continued.

The Ruszów Forest District is situated in the western part of the Bory Dolnośląskie Forest PLB020005), a continuous lowland (140–180 m a.s.l.) forest area covering 2,500 km^2^ (140–180 m a.s.l.; 51°21′N, 150°7′E). The climate is temperate, with the average annual temperature of 8.3 °C and the average annual precipitation of 550 mm^62^. The mild winters last on average for 70 days, with approximately 40 days of snow cover, an average temperature of –2 °C, and an average precipitation of 45 mm in January. The growing season (temperature over 5 °C) lasts for 220 days. The mean temperature in July, i.e. a period with the highest rainfall (100 mm), is 18 °C, compiled by^35^. It is a sparsely populated area (approx. 2,500 inhabitants within the jurisdiction of the Ruszów Forest District) with low-intensity agriculture^63^.

The principal forest tree species is the Scots pine *Pinus sylvestris*, occupying 93% of the forest area^64^. Among forest habitat types, mesic coniferous forests predominate, where birch *Betula* spp. and the common oak *Quercus robur* occur together with the Scots pine *Pinus sylvestris*, whereas bilberries *Vaccinium myrtillus*, cowberries *Vaccinium vitis-idaea*, common hairgrass *Deschampsia flexuosa*, and heather *Calluna vulgaris* commonly occur in the forest floor. Moist coniferous forests with Scots pine *Pinus sylvestris* occur in sandy areas with higher groundwater levels, where purple moor-grass *Molinia caerulea* and *Vaccinium myrtillus* predominate in the forest floor vegetation. In turn, swamp coniferous forests with wild rosemary *Ledum palustre*, bog bilberry *Vaccinium uliginosum*, and swamp cranberry *Oxycoccus palustris* occur in moister areas. Alder swamp forests and riparian forests occur only in the river valleys of the Lusatian Neisse, Czerna Mała, and Czerna Wielka rivers^63^. As a whole, coniferous forest habitats predominate in the habitat structure, as they cover approx. 94% of the area, of which moist habitats (moist mixed coniferous forest, moist coniferous forest) occupy approx. 48% of the area^64^,

### Capercaillie population

In the late 1960s, 360 Capercaillies inhabited the Bory Dolnośląskie Forest^65^. The population collapsed at the end of the 1970s, and only 200–270 birds were recorded^66^. In 2000, 11 active breeding refuges of Capercaillies were known^67^, and the inventory carried out in 2006 recorded only 18 birds in this area^68^. The last two observations of wild capercaillies (hens) in the Ruszów Forest District were recorded in 2009.

In order to re-establish the Capercaille population in the Bory Dolnośląskie Forest using the “born to be free” method^47,48,69^, 108 young Capercaillies (64 cocks and 44 hens) originating from breeding facilities of this species (Breeding Center of Forest Grouse at the Wild Park in Kadzidłowo and Capercaillie Breeding Center of the State Forests in the Wisła and Leżajsk Forest Districts) were released between 2009 and 2013 in the Ruszów Forest District. Each Capercaille received a ring with an individual number, and 62 individuals (57.4%) were monitored using VHF transmitters of the “back-pack” type with a mass of 33 g and a battery life-span of approx. 2.5 years (Biotrack Ltd.). The adaptation of the released birds to the local conditions was conducted in special adaptation areas where the Capercaillies were protected against predators (adaptation aviaries, fladry line, electric fence) and provided supplemental feeding with natural fodder^47,48^. Our observations revealed that some of the released birds began to breed in their new habitat (D. Merta, J. Kobielski, unpubl. data).

### Experimental protocols of fecal/diet analysis

Droppings were collected within the Polana Capercaillie refuge (Fig. 1). The refuge covers an area of 2,892 ha, and its boundaries were determined based on data obtained from Capercaillie telemetry monitoring between 2009 and 2013 (75% Minimum Convex Polygon) as well as historical data on the species occurrence and the distribution of lekking places.

Nearly the entire refuge area consists of coniferous forest habitats, including approximately 73% of moist habitats. The dominant species (99%) is *Pinus sylvestris*, with an admixture of *Picea abies*, *Quercus robur*, and *Betula* spp. Over 80% of the area is covered by forest stands in the III age class and older, including about 25% of stands older than 100 years. The dominant understory species are *Vaccinium myrtillus*, *Vaccinium vitis-idaea*), *Ledum palustre*, *Molinia caerulea*, and *Calluna vulgaris*. A part of the Polana refuge (an area of 255.27 ha) has functioned as a temporary protection zone since 2005, serving as a breeding ground and regular habitat for Capercaillies^70^. The forest management and the timing of the economic measures conducted in the area are subordinated to the habitat requirements of Capercaillies, which is part of the Forest Management Plan for the Ruszów Forest District for 2015–2024 and 2025–2034.

Capercaillie droppings collected over consecutive months in 2014 (May-December) and 2015 (January-April) were used in this study. The droppings were sampled from sites with a known presence of Capercaillies (Fig. 2a–g) by two co-authors of this work (D.M. and J.K.), who are experts in the biology of Capercaillies and are familiar with their appearance. The Capercaillie was the only grouse species living in the study area; hence, we excluded the possibility of confusion with other birds. Approximately 10–30 droppings were collected each month. Each fecal sample corresponds to one dropping deposited by one individual. Each dropping sample was individually labeled and stored in paper envelopes. All droppings were dried and stored at 20 °C.

To assess the dietary composition, we employed cuticle microhistological analyses of food remains found in the feces. Our assessment of the Capercaillies’ diet was an expanded version of studies conducted by us and other workers on the diet and endozoochorous seed dispersal of gallinaceous bird species with a multi-component plant diet^8,9,11,12,71,72^.

### Analysis of the diet composition

For the detailed dietary study, five or ten complete droppings were selected (magnification × 10 was used) for further processing. The analyses were conducted using a NIKON SMZ 645 stereoscopic microscope. In January, May, June, and July, at least 10 undamaged feces were available, while the number of feces analyzed in the other months was limited to five. Each feces sample was gently crushed and mixed.

The methodology employed in this study was identical to the method previously used to assess the diet of the Grey Partridge^13^ and the Yellowhammer *Emberiza citrinella*^73^, following previous dietary studies on Capercaillies^8,9,11,12,71^.

**Fig. 5:**
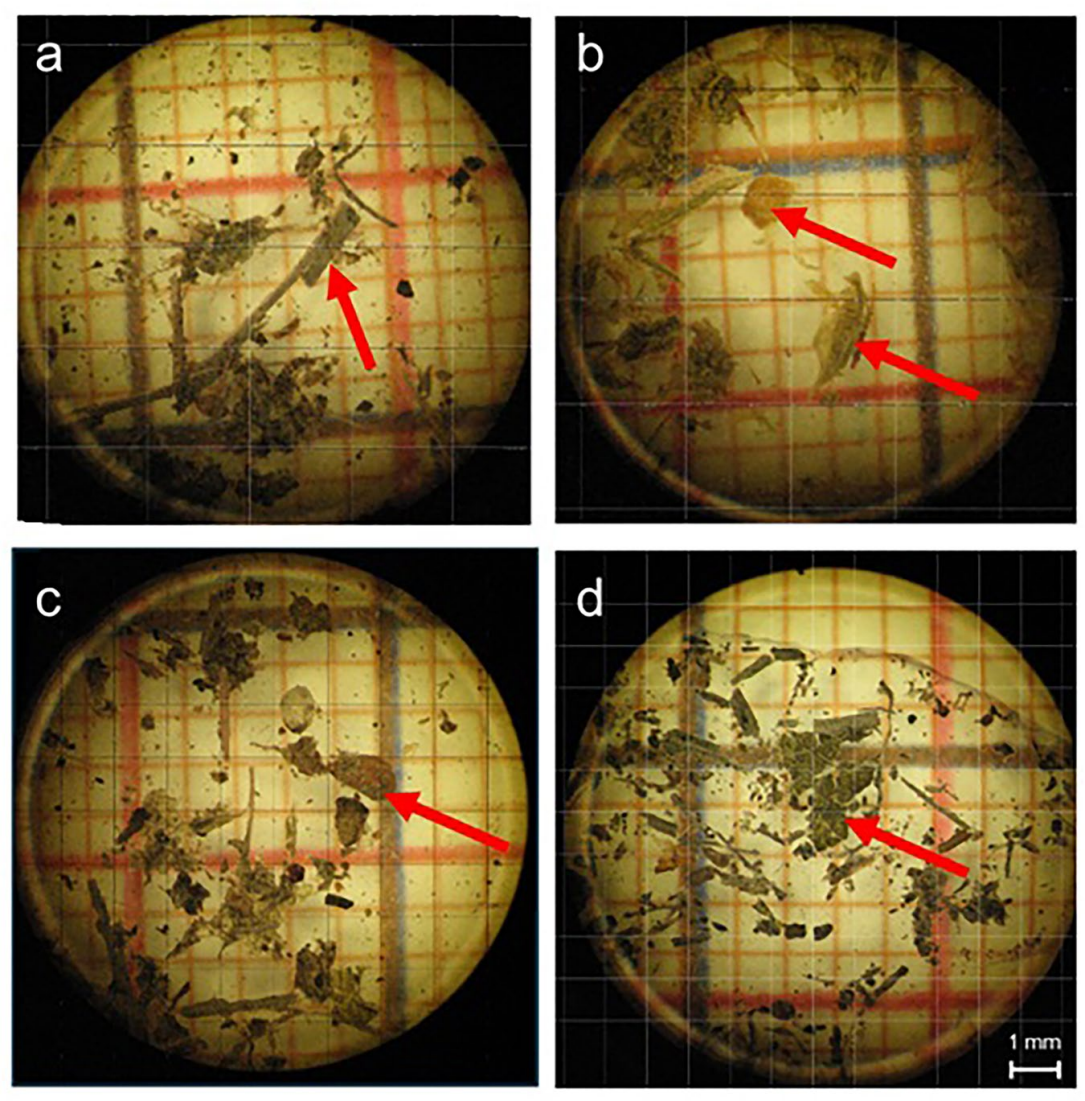
Example of microscopic examination of feces of Capercaillies: (**a**) Subsample from June; the arrow indicates a *Pinus sylvestris* needle fragment. (**b**) Subsample from June; the arrows indicate a *Carex pilulifera* seed shell and a perigynium fragment. (**c**) Subsample from July with an indicated *Vaccinium myrtillus* seed. (**d**) Subsample from July with a *Vaccinium myrtillus* leaf fragment (© Joanna Czarnecka). The red-colored grid cells are 1 mm² squares.

A 0.1-g randomly selected mixed subsample of each dropping was selected for the detailed dietary analysis. The subsamples were divided into 1-mm² squares, a total of 6,358 squares per Petri dish (Fig. 5). These squares were then examined using a stereoscopic microscope at × 40 magnification, and the number of identifiable types of plant food items was counted (Fig. 5).

Based on this examination, the approximate surface area covered by each specific type of food item was assessed (Fig. 5). The identification of the plant components of the diet was facilitated by reference materials obtained from the study area. The diet composition was expressed as the number of 49 different types of food items (42 types of plant material, 6 species of seeds and chitin fragments). The plant material was grouped into four major food categories (Table 1, Fig. 3):

1. Seed fragments and fruit remains: *Aronia* sp. pulp; *Aronia* seed shells; *Carex pilulifera* seed shells; *Carex* sp. seed shells; cereal fragments; *Juncus* sp. seed shells; *Juncus* sp. capsula fragments; *Polygonum* seed shells; *Vaccinium myrtillus* seed shells; *V. myrtillus* fruit fragments; *V. vitis-idaea* seed shells; *V. vitis-idaea* fruit fragments.
2. Fragments of leaves: *Pinus sylvestris* needles; *P. sylvestris* scale leaves; *Picea* sp. needles; *Larix* sp. needles; other unidentified needles; *V. myrtillus* leaves; V*. vitis-idaea* leaves; *Vaccinium* sp. leaves; *Fagus sylvatica* leaves; *F. sylvatica* buds; *Carex pilulifera* leaves and stems; Poaceae leaves; *Juncus* sp. leaves; unidentified dicotyledonous leaves; other leaves.
3. Moss fragments: moss leaves; fragments of moss sporangia; stem/rhizoid fragments of *Polytrichum* and *Marchantia*.
4. Other plant material: *Pinus sylvestris* cone fragments; *Vaccinium* sp. stamen fragments; *Vaccinium* sp. stem fragments; cereal husks; *Calamagrostis* sp. husks; *Poa compressa* husks; other Poaceae husks; *P. compressa* inflorescence fragments; *Carex pilulifera* inflorescence fragments; *C. pilulifera* perigynium fragments; twig fragments; unidentified plant material.

The dataset also includes information on the number of stones (presumably originating from birds foraging on forest roads; Fig. 2d) and minor animal food (see below), based solely on a rough inspection of sclerotized fragments of arthropods (chitin fragments).

The amount of the above food items was given per 0.1 g of feces (Table 1, Fig. 3).

### Analysis of the seed content

After collecting the subsamples (0.1 g for each fecal sample), the rest of the fecal material was analyzed under × 10 magnification to assess the number of intact ( = endozoochorically transferred) seeds. Additionally, the intact seeds were counted in the 0.1-g subsamples tested to assess the diet. The information on the number of intact seeds in the feces is given in Table 1 (separately for whole samples and subsamples). In the graph (Fig. 4), the number and proportion of intact seeds per 1 g of feces are given to facilitate comparisons between the months of sample collection.

### Analysis of the contribution of arthropods

Additionally, to evaluate the contribution of arthropods to the diet of Capercaillies, which adult birds occasionally eat between spring and summer^59^, we conducted a detailed examination of a large sample of 61 droppings collected in May 2014 (*n* = 28 droppings) and June 2014 (*n* = 33 droppings) (Table 3). This assessment was an expanded version of our previous dietary studies on invertebrate-feeding birds based on fecal analysis^74,75^. Briefly, the identification of the arthropod components was performed under a stereoscopic microscope ( × 20), after separation in Petri dishes. The number of prey items representing particular invertebrate taxa was established based on the quantity of fragments of chitin parts, chiefly the elytra (Coleoptera and Heteroptera), mouthparts (Araneae), and other preserved organs (e.g. limbs, petiolus, clypeus, or mandibule). When determining the number of prey belonging to particular taxa, we applied a rule of summation of different chitin parts to the level of one individual, i.e. two or more different fragments of chitin parts (e.g. head, mandibles, six legs, and other parts in the case of ants) from one dropping was treated as belonging to the same individual of a given species.

## Data Records

The dataset presented in Tables 1, 3 is stored and available for downloading at the Zenodo data repository^76^ 10.5281/zenodo.15510661. Due to the large number of zero values for certain individual food items, the numerical data from Tables 1, 3 were compiled only for items recorded in the examined fecal samples. The dataset available at the Zenodo data repository^76^ is in an editable and tabulated format summarized in two Excel sheets: ‘Plant component’ (containing the complete list of food items for consecutive months) and ‘Arthropod Component’. Table 1 contains raw data on the number of different food items identified in the examined droppings. The first column in the dataset describes plant components identified to the highest most possible level. The plant identification (the first column) is provided through scientific Latin names and the morphological characteristic of the plant part (tissue). The information on the number of small stones (grit) and the animal component (chitin parts) is provided in the last rows of Table 1. The next 80 columns (B-CC) contain information on the total weight and the number of different food items recorded in each dropping (*n* = 80).

## Technical Validation

The description of the multi-component plant diet was based on taxon- and morpho-specific classifications of food items aligned with previous dietary studies on Capercaillies^8,9,11,12,71^. We recorded the names of prey items with as much taxonomic resolution as the visual inspection allowed, following the prior results published in peer-reviewed papers. Due to the use of the previous nomenclature describing the composition of the Capercaillies’ diet, it is assumed that the quality of our methodology and analysis meet the criteria of reproducibility and comparability with other studies. Considering the differences in habitat conditions regarding food availability, the diet of Capercaillies in our study sites may show different phenology and related contribution of specific plant components.

## Usage Notes

Our study addresses a critical gap in herbivore diet studies by emphasizing the importance of identifying morphologically distinct plant parts, which molecular methods often overlook. The detailed description of sample collection, processing, and analysis procedures ensures the reproducibility of the research.

By providing the integrative quantitative dietary data based on the functional classification of various plant parts, representing 49 different plant food items from four major food categories (which can be regarded as dietary correlates), as well as intact seeds, arthropods, and mineral particles (grit), our dataset has potential applications in studies of the diet, dispersal capabilities, and the reintroduction biology of gallinaceous birds.

Our dietary data, derived from microhistological analyses, strongly contrast with some recently published dietary data for herbivorous birds, including Galliformes. Specifically, the existing databases and certain published papers (primarily those based on molecular techniques, e.g., DNA metabarcoding) on bird diets defined by the authors themselves as “quantitative”, do not contain sufficient numeric information on the various plant parts consumed and only present the taxonomical composition of the diet/fecal sample^6,7,77^. This limitation significantly restricts the practical application of these data in understanding the biological characteristics or habitat requirements of specific species in the context of plant-animal interactions and in formulating recommendations for the conservation and management of Galliformes in variable habitats. It should be emphasized, however, that several earlier studies have thoroughly described the diets of Galliformes, including the Capercaillie, by considering the morpho-histological distinctions of different plant parts^8–12^, and our methodology was based on these previous approaches.

Our dataset did not contain information on the germinability of *Vaccinium myrtillus* seeds recovered from the Capercaillie droppings; however, a prior study showed that the survival rate of such seeds (determined by indigocarmine stain tests) was less than 2%^16^. By providing data on the taxon- and morpho-specific distinctions of various plant parts, the presented dataset complements our previous observations of the major plants ingested by the Capercaillies in our study area^35^. In our earlier dietary study, based on field observations of foraging Capercaillies, we were only able to identify the plant taxa consumed without specifying which parts of the plants were eaten^35^. Notably, the current dataset enables us to differentiate between two berry species that were previously grouped together: *Vaccinium myrtillus* and *V. vitis-idaea* (the latter of which was rarely consumed in late autumn; see Table 1). Additionally, it provides a precise description of parts of the plants (primarily from coniferous trees) that were consumed (Table 1).

It is important to recognize that Galliformes consume various fragments of the same plant taxa with differing intensity throughout the year. Consequently, any qualitative methods relying on the frequency of occurrence of individual plant taxa based on fecal DNA do not yield accurate quantitative data on the diet composition in terms of the specific plant parts ingested. Therefore, such methods should be rather avoided in dietary studies of herbivores that specialize in consuming specific plant fragments. Hence, a parallel microhistological analysis is essential to describe the full spectrum of the diet of herbivores consuming various plant fragments.

## Data Availability

We did not use a custom code for the data management.
